# Subjective Outcome Evaluation Findings: Factors Related to the Perceived Effectiveness of the Tier 2 Program of the Project P.A.T.H.S.

**DOI:** 10.1100/tsw.2010.19

**Published:** 2010-02-12

**Authors:** Daniel T.L. Shek, Cecilia M.S. Ma

**Affiliations:** ^1^ Department of Applied Social, Sciences Hong Kong Polytechnic University, Hong Kong; ^2^ Public Policy Research Institute, Hong Kong Polytechnic University, Hong Kong; ^3^ Kiang Wu Nursing College of Macau, Macao

**Keywords:** subjective outcome evaluation, positive youth development program, volunteer training and services, adventure-based counseling

## Abstract

After completion of the Tier 2 Program of the Project P.A.T.H.S. (Positive Adolescent Training through Holistic Social Programmes), 8,489 participants in 196 schools responded to the Subjective Outcome Evaluation Form (Form C) to assess their views of the program, program workers, and perceived effectiveness of the program. Four major program elements were identified, including programs based on the adventure-based counseling approach (n = 48), programs concentrated on volunteer training and services (n = 44), programs with both the adventure-based counseling approach and volunteer training activities (n = 63), and other programs with different foci (n = 41). Descriptive statistics showed that the respondents had positive perceptions of the program, workers, and benefits of the program. Perceived qualities of the program and the program workers were positively associated with perceived effectiveness of the program. Multiple regression analysis revealed that perceived qualities of the program, but not the program workers, predicted perceived effectiveness of the program. The theoretical and practical implications of the findings are discussed.

## INTRODUCTION

Experiential education refers to “learning by doing” ([1], p. 5). The value of using experiential activities with a variety of client concerns and modalities is well documented[2,3,4,5]; in particular, the adventure-based counseling (ABC) technique. It has long been used extensively in clinical[6,7] and school settings[8], and is a strength-based, adventure-based approach to personal development and therapeutic activity. ABC aims to incorporate action and learning in order to promote positive change, such as self-concept, self-efficacy, and interpersonal skills, through outdoor activities[9]. Evaluation studies showed that ABC programs are beneficial in helping at-risk youth[10,11].

To date, most of the youth programs have involved one program type only. Rapee and associates[12] demonstrated the association of program satisfaction and stigma, and how this relationship differs by modes of program delivery (i.e., universal vs. indicated programs) among at-risk youth. Based on an extensive review of the literature, Nation and colleagues[13] identified that varied teaching methods and comprehensiveness are critical principles associated with effective youth prevention programs. Given the lack of comparison of program components in the positive youth development literature, the aims of the present study are to examine the factors associated with program effectiveness and how these relationship(s) differ by the program elements.

Advocates of the positive youth development approaches argue that "problem free is not fully prepared" for youths to enter healthy and satisfying adulthood[14,15], and they urge a focus on youth strengths rather than viewing them as problems[16]. Among the positive youth development programs that have undergone empirical investigation, few studies are conducted in the Chinese context. Furthermore, little is known about the factors that are associated with the program effectiveness among Chinese adolescents.

The Project "P.A.T.H.S. to Adulthood: A Jockey Club Youth Enhancement Scheme" is a large-scale positive youth development program designed for junior secondary school students (Secondary 1 to 3, i.e., Grades 7 to 9) in Hong Kong[17]. The word "P.A.T.H.S." denotes **P**ositive **A**dolescent **T**raining through **H**olistic **S**ocial **P**rogrammes. It consists of two tiers of program. The Tier 1 Program targets all students who join the program in a particular form (i.e., universal prevention initiative). Through the use of structured curriculum, students learn competencies with reference to the 15 positive youth development constructs[17]. The Tier 2 Program is specially designed for students with greater psychosocial needs in different psychosocial domains (i.e., selective prevention). It is noteworthy that the researchers of this project deliberately avoided using the term "at risk" because the term is very stigmatizing in the Chinese culture, and it deters parents and students from joining the related programs. Based on the developmental needs of the students in a particular school, the school social work service providers design and implement the Tier 2 Program[18,19].

The primary purpose of this study was to report and discuss the findings of an evaluation of the Tier 2 Program. In addition, as there is sparse literature on the types of program elements that are most effective for positive youth development, especially in the Chinese context, the present study attempted to fill the gap by examining the factors related to the effectiveness of a positive youth development program in Hong Kong. Furthermore, it explored whether these relationships differ by the program elements based on the perspectives of the participants. It was hoped that the result of the present study would help practitioners to understand what types of program elements facilitate positive youth development when designing programs for this population.

## METHODS

### Participants

A total of 196 schools joined the Project P.A.T.H.S. (Secondary 2 Level) in the second year of the Full Implementation Phase in the 2007/08 school year. In these schools, there were 12,490 participants involved in the Tier 2 Program. Among them, 11,347 students were identified by teachers, parents, and/or via self-administered questionnaires as having greater psychosocial needs and they were invited to join the Tier 2 Program. Additionally, a total of 1,143 participants who are the parents and teachers of those identified students also joined the Tier 2 Program. The mean number of participants joining the Tier 2 Program per school was 63.72 (range: 10–435). The average number of sessions provided per school (normally 1.5–3 h per session) was 23.13 (range: 5–119).

The participants were invited to respond to the Subjective Outcome Evaluation Form (Form C) developed by the research team[20], usually immediately after completion of the Tier 2 Program. A total of 8,489 participants (mean = 43.29 participants per school; range: 7–196) responded to Form C and the overall response rate was 67.97%. There are three plausible reasons for this low response rate: (1) the participants withdrew from the Tier 2 Program before completion, (2) some participants were absent from the last session and did not complete the evaluation form, or (3) some schools did not invite the adult participants to respond to the evaluation form.

## Procedures

At the beginning of data collection, the purpose of the evaluation was explained and the principle of confidentiality was repeatedly emphasized to the participants. The participants were asked to indicate their wish if they did not want to respond to the evaluation questionnaire (i.e., "passive" informed consent was obtained). All participants responded to all scales in the evaluation form in a self-administration manner. Adequate time was provided for the participants to complete the questionnaire. To facilitate the program evaluation, the research team developed an evaluation manual with standardized instructions for collecting the subjective outcome evaluation data[20]. In addition, adequate training was provided to the social workers during 20-h training workshops on how to collect and analyze the data collected using Form C.

## Instruments

The Subjective Outcome Evaluation Form (Form C), designed by Shek et al.[20], aims to measure the perceptions of the Tier 2 Program. There are seven parts in this evaluation form:

1. Participants' perceptions of the program, such as program design, quality of service, appropriateness of the program, and interaction among the participants (eight items).
2. Participants' perceptions of the workers, such as the preparation of the workers, professional attitude and knowledge, and interaction with the participants (eight items).
3. Participants' perceptions of the program effectiveness, such as promotion of different psychosocial competencies, resilience, and overall personal development (seven items). Item 3 (i.e., "In the future, participants would receive similar service(s) if needed.") was removed as we were only interested in participants' perceived changes after the participation of the program.
4. Things that the participants appreciated most (open-ended question).
5. Opinion about the workers (open-ended question).
6. Things that the participants learned from the program (open-ended question).
7. Areas that require improvement (open-ended question).

After collecting the data, the social work service providers were requested to input the data in an EXCEL file developed by the research team that would automatically compute the frequencies and percentages associated with the different ratings for an item. When the providers submitted the reports, they were also requested to submit the soft copy of the consolidated datasheets. The data from all service providers were then aggregated to "reconstruct" the overall profile by the research team. Since some amendments were made during the program implementation (e.g., program cancellation due to insufficient participants), the delivered program content was reported again in the program report.

Since the program contents of the Tier 2 Program were designed according to the various needs and the contextual situations of different schools, it is hard to compare their results directly. Therefore, an analysis on the program contents was conducted to identify the major program elements of the Tier 2 Program for all participating schools. Three trained research assistants (one with a Bachelor's degree in Psychology, one with a Bachelor's degree in Social Sciences, and one with a Master's degree and 3 years front-line social work experience) categorized the program contents delivered by the social workers as indicated in the program reports submitted to the research team. The criteria were strictly set to categorize a program according to the program proposal or revised proposal together with the actual activities.

### Data Analyses

Percentage findings were examined using descriptive statistics. A composite measure of each factor (i.e., perceived qualities of program content, perceived qualities of program workers, and perceived program effectiveness) was created based on the total scores of each factor divided by the number of items. Pearson correlation was used to examine if the program content and program workers were related to the program effectiveness. A hierarchical linear regression was performed to compare which factor would predict the program effectiveness. All analyses were performed by using the Statistical Package for Social Sciences Version 17.0[21].

## RESULTS

Adopting the same methodology used in previous studies (e.g., [18]), two major types of program elements (i.e., the ABC approach and volunteer training and services) were identified. A program will be classified as containing the ABC elements if and only if the espoused theory was matched with the theory-in-action[22]. The most frequently found activities related to volunteer training and services (VTS) were program planning skills and organizing volunteer services. The most popular volunteer services were visiting the elderly and services for mentally challenged people. The programs without elements of ABC or VTS were classified as “others”. Activities in this program included groups promoting self-understanding, social skills training, and community-based oral history projects.

Tables 1–4 summarize the program characteristics of the Tier 2 Program. Most of the schools (32.1%) adopted the program that consisted of both the ABC approach and VTS elements. There were 24.5 and 22.4% of the schools that selected the ABC element only and VTS element only, respectively. Lastly, 20.9% of the schools adopted other programs. Percentage data showed that most of the participants had positive perceptions of the program, participants, and benefits of the program.

Descriptive statistics, correlations coefficients, Cronbach’s alphas, and mean of interitem correlations are shown in Table 5. By using the schools as the units of analyses, the internal consistency of each factor was high (i.e., above 0.95). Results of correlation analyses showed that both program content ($r = 0.89$, $p < 0.01$) and program workers ($r = 0.83$, $p < 0.01$) were strongly associated with program effectiveness.

Table 6 presents multiple regression analysis results. Higher positive views toward the program were associated with higher program effectiveness ($\beta = 0.87$, $p < 0.01$). However, perception on program workers was not significantly related to program effectiveness ($\beta = 0.01$, $p > 0.01$). This model explained 78% of the variance toward the prediction of program effectiveness. Interestingly, these findings were consistent across program types. This indicated that, regardless of program types, perceived positive views toward the program, but not perceived qualities of program workers, were significantly related to program effectiveness. The amount of variance toward the prediction of program effectiveness was similar across program types, ranging from 0.76 to 0.88.

## DISCUSSION

The present study examined the relationship between program characteristics and program effectiveness in Chinese positive youth programs; particularly, it explored whether this relationship differs by program types. Consistent with the observations commonly found in subjective outcome evaluation studies, a major proportion of the respondents perceived the program, workers, and effectiveness in a favorable light. Of course, there were negative responses and they can help to improve the program in future.

**TABLE 1**
**Summary of Program Characteristics and Effectiveness**

| Main Program Approach | Clientele | Average No. of Participants | Average Program Attendance (%) | Average No. of Program Aims Indicated in the Reports | Average No. of Constructs Indicated in the Reports | Mean of Overall Effectiveness |
|---|---|---|---|---|---|---|
| ABC and VTS[1] (n = 63, 32.1%) | a (n = 54) | 42.17 | 80.02 | 2.65 | 7.04 | 4.60 |
| | b (n = 7) | 68.14 | 87.50 | 2.43 | 7.14 | 4.67 |
| | c (n = 1) | 60.00 | 80.00 | 3.00 | 8.00 | 4.73 |
| | d (n = 1) | 365.00 | 95.00 | 2.00 | 10.00 | 4.44 |
| ABC[2] (n = 48, 24.5%) | a (n = 42) | 53.88 | 83.65 | 2.00 | 6.62 | 4.57 |
| | b (n = 3) | 99.33 | 88.18 | 1.33 | 5.00 | 4.49 |
| | c (n = 1) | 182.00 | 86.00 | 2.00 | 7.00 | 4.47 |
| | d (n = 2) | 63.50 | 91.80 | 3.00 | 5.50 | 4.81 |
| VTS[3] (n = 44, 22.4%) | a (n = 39) | 49.85 | 81.45 | 2.51 | 6.38 | 4.61 |
| | b (n = 5) | 58.40 | 81.52 | 2.20 | 5.40 | 4.45 |
| Others[4] (n = 41, 20.9%) | a (n = 31) | 51.55 | 77.92 | 1.90 | 6.61 | 4.48 |
| | b (n = 10) | 98.10 | 83.38 | 2.20 | 5.70 | 4.57 |

*Note:* a = Only students involved; b = students and parents involved; c = students and teachers involved; d = students, parents, and teachers involved.

1 The program contents related to both the ABC approach and VTS were indicated in the Tier 2 Program reports.

2 The program contents related to the ABC approach were indicated in the Tier 2 Program reports.

3 The program contents related to VTS were indicated in the Tier 2 Program reports.

4 Except for the ABC approach and VTS, other program contents were indicated in the Tier 2 Program reports.

Several points can be highlighted in the study. First, two programs, i.e., the ABC only and the VTS only, each took up about 22–24% of all programs, but a combination of the ABC approach and VTS only program took up 32%. These two program approaches covered 79% of all delivered programs and were the dominant approaches adopted. They were both popular program approaches used by social workers working with adolescents and young people in Hong Kong. Both of them ground their theoretical base in experiential learning theory[23,24,25]. The VTS element only program has a much longer history that can date back to the late 1960s and has since been a popular program, while the ABC only program was formally adopted as the major program theory for a huge social intervention program entitled “The Understanding the Adolescent Project” (UAP) to combat problems among students identified as adolescent-at-risk from 2001 to 2004 in Hong Kong[26,27].

## TABLE 2
## Comparison of the Positive Views towards the Tier 2 Program among Different Program Approaches

| | Participants with Positive Responses in Different Program Approaches | | | | | | | | | |
|---|---|---|---|---|---|---|---|---|---|---|
| | ABC and VTS[1] | | ABC[2] | | VTS[3] | | Others[4] | | Overall | |
| | n (Total Response) | % | n (Total Response) | % | n (Total Response) | % | n (Total Response) | % | n (Total Response) | % |
| The activities were carefully planned. | 2,107 (2,390) | 88.16 | 2,044 (2,333) | 87.61 | 1,657 (1,916) | 86.48 | 1,531 (1,789) | 85.58 | 7,339 (8,428) | 87.08 |
| The quality of the service was high. | 2,122 (2,386) | 88.94 | 2,026 (2,332) | 86.88 | 1,656 (1,914) | 86.52 | 1,512 (1,787) | 84.61 | 7,316 (8,419) | 86.90 |
| The service provided could meet the participants' needs. | 2,115 (2,387) | 88.60 | 2,039 (2,327) | 87.62 | 1,657 (1,909) | 86.80 | 1,527 (1,784) | 85.45 | 7,338 (8,410) | 87.25 |
| The service delivered could achieve the planned objectives. | 2,128 (2,389) | 89.07 | 2,052 (2,321) | 88.41 | 1,669 (1,911) | 87.34 | 1,507 (1,785) | 84.43 | 7,356 (8,406) | 87.51 |
| Participants could get the service they wanted. | 2,079 (2,387) | 87.10 | 1,985 (2,324) | 85.41 | 1,607 (1,912) | 84.05 | 1,449 (1,784) | 81.22 | 7,120 (8,407) | 84.69 |
| Participants had much interaction with other participants. | 2,134 (2,386) | 89.44 | 2,061 (2,323) | 88.72 | 1,674 (1,907) | 87.78 | 1,527 (1,782) | 85.69 | 7,396 (8,398) | 88.07 |
| Participants would recommend others who have similar needs to participate in the program. | 2,002 (2,388) | 83.84 | 1,947 (2,320) | 83.92 | 1,555 (1,911) | 81.37 | 1,442 (1,784) | 80.83 | 6,946 (8,403) | 82.66 |
| On the whole, participants were satisfied with the service. | 2,159 (2,383) | 90.60 | 2,096 (2,324) | 90.19 | 1,683 (1,910) | 88.12 | 1,567 (1,783) | 87.89 | 7,505 (8,400) | 89.35 |

[1–4] See footnotes for Table 1.

## TABLE 3
## Comparison of the Positive Views towards the Tier 2 Program Workers among Different Program Approaches

| | Participants with Positive Responses in Different Program Approaches | | | | | | | | | |
|---|---|---|---|---|---|---|---|---|---|---|
| | ABC and VTS[1] | | ABC[2] | | VTS[3] | | Others[4] | | Overall | |
| | n (Total Response) | % | n (Total Response) | % | n (Total Response) | % | n (Total Response) | % | n (Total Response) | % |
| The worker(s) had professional knowledge. | 2,214 (2,390) | 92.64 | 2,114 (2,330) | 90.73 | 1,738 (1,915) | 90.76 | 1,610 (1,787) | 90.10 | 7,676 (8,422) | 91.14 |
| The worker(s) demonstrated good working skills. | 2,183 (2,388) | 91.42 | 2,111 (2,332) | 90.52 | 1,734 (1,913) | 90.64 | 1,574 (1,786) | 88.13 | 7,602 (8,419) | 90.30 |
| The worker(s) were well prepared for the program. | 2,201 (2,381) | 92.44 | 2,134 (2,325) | 91.78 | 1,757 (1,909) | 92.04 | 1,625 (1,785) | 91.04 | 7,717 (8,400) | 91.87 |
| The worker(s) understood the needs of the participants. | 2,166 (2,388) | 90.70 | 2,090 (2,324) | 89.93 | 1,705 (1,909) | 89.31 | 1,571 (1,787) | 87.91 | 7,532 (8,408) | 89.58 |
| The worker(s) cared about the participants. | 2,190 (2,384) | 91.86 | 2,128 (2,329) | 91.37 | 1,729 (1,912) | 90.43 | 1,603 (1,787) | 89.70 | 7,650 (8,412) | 90.94 |
| The worker(s)' attitudes were very good. | 2,195 (2,384) | 92.07 | 2,117 (2,323) | 91.13 | 1,750 (1,914) | 91.43 | 1,596 (1,782) | 89.56 | 7,658 (8,403) | 91.13 |
| The worker(s) had much interaction with participants. | 2,129 (2,388) | 89.15 | 2,051 (2,324) | 88.25 | 1,672 (1,915) | 87.31 | 1,522 (1,783) | 85.36 | 7,374 (8,410) | 87.68 |
| On the whole, participants were satisfied with the worker(s). | 2,190 (2,387) | 91.75 | 2,134 (2,329) | 91.63 | 1,763 (1,914) | 92.11 | 1,627 (1,788) | 91.00 | 7,714 (8,418) | 91.64 |

[1–4] See footnotes for Table 1.

## TABLE 4
## Comparison of the Positive Views towards the Tier 2 Program Effectiveness among Different Program Approaches

| | Participants with Positive Responses in Different Program Approaches | | | | | | | | | |
|---|---|---|---|---|---|---|---|---|---|---|
| | ABC and VTS[1] | | ABC[2] | | VTS[3] | | Others[4] | | Overall | |
| | n (Total Response) | % | n (Total Response) | % | n (Total Response) | % | n (Total Response) | % | n (Total Response) | % |
| The service helped participants a lot. | 2,060 (2,366) | 87.07 | 1,977 (2,303) | 85.84 | 1,601 (1,896) | 84.44 | 1,480 (1,762) | 84.00 | 7,118 (8,327) | 85.48 |
| The service enhanced participants' growth. | 2,096 (2,362) | 88.74 | 2,014 (2,304) | 87.41 | 1,633 (1,896) | 86.13 | 1,502 (1,761) | 85.29 | 7,245 (8,323) | 87.05 |
| In the future, participants would receive similar service(s) if needed. | 2,024 (2,362) | 85.69 | 1,946 (2,303) | 84.50 | 1,592 (1,891) | 84.19 | 1,434 (1,761) | 81.43 | 6,996 (8,317) | 84.12 |
| Participants learned how to help themselves through participating in the program. | 2,110 (2,359) | 89.44 | 2,033 (2,297) | 88.51 | 1,652 (1,890) | 87.41 | 1,539 (1,758) | 87.54 | 7,334 (8,304) | 88.32 |
| Participants had positive change(s) after joining the program. | 2,087 (2,361) | 88.39 | 1,982 (2,303) | 86.06 | 1,635 (1,890) | 86.51 | 1,500 (1,758) | 85.32 | 7,204 (8,312) | 86.67 |
| Participants learned how to solve their problems through participating in the program. | 2,095 (2,346) | 89.30 | 2,028 (2,292) | 88.48 | 1,636 (1,888) | 86.65 | 1,508 (1,749) | 86.22 | 7,267 (8,275) | 87.82 |
| Participants' behavior became better after joining this program. | 1,978 (2,356) | 83.96 | 1,917 (2,299) | 83.38 | 1,528 (1,890) | 80.85 | 1,400 (1,760) | 79.55 | 6,823 (8,305) | 82.16 |
| Those who knew the participants agreed that this program induced positive changes in them. | 1,978 (2,355) | 83.99 | 1,888 (2,292) | 82.37 | 1,527 (1,892) | 80.71 | 1,431 (1,752) | 81.68 | 6,824 (8,291) | 82.31 |

[1–4] See footnotes for Table 1.

## TABLE 5
## Mean, Standard Deviations, Correlation Coefficients, Cronbach's Alphas, and Mean of Interitem Correlations among Subjective Outcome Evaluation Measures

| | 1 | 2 | 3 | M | SD | α | Mean Interitem Correlations |
|---|---|---|---|---|---|---|---|
| Perceived qualities towards the Tier 2 Program (eight items) | — | | | 4.62 | 0.36 | 0.98 | 0.87 |
| Perceived qualities towards the Tier 2 Program workers (eight items) | 0.91** | — | | 4.83 | 0.35 | 0.97 | 0.84 |
| Perceived Tier 2 Program effectiveness (seven items)[a] | 0.89** | 0.83** | — | 4.57 | 0.36 | 0.98 | 0.89 |
| Total (23 items) | — | — | — | 4.68 | 0.14 | 0.99 | 0.81 |

[a] Item 3 was removed (i.e., "In the future, participants would receive similar service(s) if needed").

** $p < 0.001$.

**TABLE 6**
**Multiple Regression Analyses Predicting Positive Views towards the Tier 2 Program Effectiveness**

| | Tier 2 Program Content | Tier 2 Program Workers | |
|---|---|---|---|
| | β[a] | β[a] | $R^2$ |
| ABC and VTS[1] | 0.81** | 0.08 | 0.79 |
| ABC[2] | 0.69** | 0.20 | 0.76 |
| VTS[3] | 0.90** | –0.03 | 0.76 |
| Others[4] | 0.89** | 0.05 | 0.88 |
| Overall | 0.87** | 0.01 | 0.78 |

[1–4] See footnotes for Table 1.

[a] Standardized coefficients.

** $p < 0.01$ (0.01/5 = Bonferroni-corrected alpha level).

It is noteworthy that although a combination of the ABC approach and VTS, as well as VTS only, are popular programs offered by social workers in Hong Kong, the effectiveness of these programs has not been properly documented. Although the ABC approach can help to instill novel experiences in young people who are usually not good at verbal expression (i.e., walk therapy rather than talk therapy) and its usefulness has been endorsed by counselors[6,8], its long-term effect is questionable[25]. The positive evaluation findings based on the service learning programs are basically consistent with the literature that engagement in voluntary service is conducive to the positive development of adolescents[28,29].

The data from this study showed that positive perception on program content was related to program effectiveness. In conjunction with review studies[13,30,31,32], perceived qualities of program content, especially with the inclusion of multiple settings and domains such as family, school, and community, was associated with program effectiveness. One of the characteristics of the Tier 2 Program is the use of a highly structured approach, the provision of a wide variety of activities, and the involvement of adults as the major components of the Tier 2 Program. It invited the participation of parents and teachers in school and community settings. Nation et al.[13] noted that “Programs that engage children/adolescents and their environment context are most likely to produce change (p. 455)”. Examples of the activities in the Tier 2 Program included (a) mentorship programs involving the alumni of the schools, (b) mental health promotion programs, (c) adventure-based counseling, (d) parenting programs, (e) service learning programs, and (f) resilience enhancement programs. Researchers highlighted the importance of structured program activities, context for establishing supportive relationship with adults, and tailored to the sociocultural needs of the participants for effective positive youth development program[16,33].

In addition, the design of the Tier 2 Program provides implementers and workers the flexibility to design appropriate programs that target the needs of the students with reference to the positive youth development constructs[17]. In other words, the Tier 2 Program activities were able to address the unique and specialized needs of students. Nixon[34] noted that “Young people…need supports, opportunities, and services provided by adults who recognize and respect each youth’s ability to take an active role” (p. 572). The activities in the Tier 2 Program not only aimed to foster greater involvement of family and community members, but also provided opportunities to these greater psychological needs students to explore their identity[35], develop inter-relatedness with others, and reduce the risk of engaging deviant or risky behavior. These activities offer them opportunities to model prosocial behaviors, strengthen the bond with parents and teachers via parental involvement in family activities, and facilitate the interaction with other community members and nonprofit organization personnel. Evidence demonstrated that participation in voluntary service has a beneficial influence on adolescents’ psychological well-being in Western[28,29] and Chinese contexts[36]. As the Tier 2 programs varied in their content and context, this might help to explain the absence of variation among program types regarding the relationship between program content and program effectiveness. The present findings shed light on the design and implementation of effective positive youth development programs.

Contrary to prior studies[37,38], perceived qualities of program workers was not a significant predictor to program effectiveness. Some might question whether this is related to the quality of program workers, as well-trained staff is one of the important factors contributed to program effectiveness[13]. It is noteworthy that all workers in the Tier 2 Program are experienced front-line social workers who have at least 3 years of experience in working with youths, and they have attended relevant training workshops for more than 20 h. One possible explanation for the absence of relationship may be related to the highest mean score of perceived qualities of workers ($M$ = 4.83, $SD$ = 0.35) as compared to the other factors (perceived qualities of program content: $M$ = 4.62, $SD$ = 0.36; perceived program effectiveness: $M$ = 4.57, $SD$ = 0.36) (Table 5). Further, about 90% of the participants, regardless of program types, were satisfied with the program workers (Table 3).

A previous study demonstrated that this ceiling effect might be a plausible source of nonsignificant results[39]. The high rating of perceived qualities of workers might be a viable alternative reason of this phenomenon. Perhaps future study should be conducted to explore whether other factors (e.g., satisfaction towards the program, relationship with program workers) would mediate/moderate this relationship in order to provide a more comprehensive picture in this issue.

There are several limitations in the present study. First, the data were based on self-report measures. Further studies are needed to evaluate the program by different approaches (e.g., focus group interviews, diaries, and process evaluation) and sources (e.g., teachers, social workers, parents). Second, as the present findings were “reconstructed” from the evaluation reports submitted by the agencies, the units of analyses were schools, instead of individuals. Therefore, the power of statistical analyses would become low and individual variations lost in the process. Evidently, analyses based on data collected from each participant should be attempted in the future. Third, the Tier 2 Program aimed at creating positive changes among adolescents with greater psychological needs. It provided opportunities for participants to transfer their experiences from the experiential activities back into their daily lives. Program implementers attempted to develop rapport between students and program workers, tailor activities to meet the specific needs of these adolescents with greater psychosocial needs, and create an environment of trust and support among participants. Therefore, the program content was not strictly framed in order to allow the program implementers to customize the program activities flexibly without restrictions. More research is warranted to examine how well this factor affects the program effectiveness in positive youth development programs.

Despite the aforementioned limitations, this study makes several contributions to the positive youth development literature. First, it reveals that there is a significant association between the positive perception of the program and program effectiveness. To date, very few studies have examined what factors contributed to the program effectiveness. The present findings provide insights to practitioners when designing appropriate positive youth programs in the Chinese context.

Second, this study has provided some initial quantitative evidence that what factors are associated with perceived program effectiveness, particularly, there is a paucity of experiential education research among Chinese adolescents with greater psychological needs. Shek[40] argued that more research work is needed on subjective outcome evaluation, especially in social work education. The findings of the present study can be viewed as a constructive response to the existing social work practice literature.

## ACKNOWLEDGMENTS

The preparation for this paper and the Project P.A.T.H.S. were financially supported by The Hong Kong Jockey Club Charities Trust.

## References

1. Lewis, L. and Williams, C.J. (1994) Experiential learning: past and present. In *Experiential Learning: A New Approach.* Jackson, L. and Caffarella, R.S., Eds. Jossey-Bass, San Francisco. pp. 5–16.
2. Blatner, A. (2005) Psychodrama. In *Current Psychotherapies*. Corsini, R.J. and Wedding, D., Eds. Thomson Brooks/Cole Publishing, Belmont, CA. pp. 405–438.
3. Glass, J. (2006) Working toward aesthetic distance: drama therapy for adult victims of trauma. In *Expressive and Creative Arts Methods for Trauma Survivors*. Carey, L.J., Ed. Jessica Kingsley Publishers, London. pp. 57–71.
4. Malchiiodi, C. (2008) *Creative Interventions with Traumatized Children*. Guilford Press, New York.
5. Oaklander, V. (2001) Gestalt play therapy. *Int. J. Play Ther.* **10(2),** 45–55.
6. Fletcher, T. and Hinkle, J.S. (2002) Adventure based counseling: an innovation in counseling. *J. Couns. Dev*. **80**, 277–285.
7. Gilliam, N. (1993) Foundations of adventure therapy. In *Adventure Therapy*. Gass, M.A., Ed. Kendall Hunt, Dubuque, IA. pp. 3–10.
8. Glass, J.S. and Myers, J.E. (2001) Combining the old and the new to help adolescents: individual psychology and adventure-based counseling. *J. Ment. Health Couns.* **23(2),** 104–114.
9. Clements, C. and Wagner, R. (1995) The ins and outs of experiential training. *Training Dev*. **49(2),** 52–56.
10. Lieberman, M.A. and DeVos, F. (1982) ABC Final Evaluation Report. September 1982. MA Project Adventure, Hamilton.
11. Sakofs, M. and Schuurman, D. (1991) Assessing the Impact of the Wilderness Alternative for Youth Program: an Outward Bound Program for Adjudicated Youth. Outward Bound, Greenwich, CI.
12. Rapee, R.M., Wignall, A., Sheffield, J., Kowalenko, N., Davis, A., McLoone, J., and Spence, S.H. (2006) Adolescents' reactions to universal and indicated prevention programs for depression: perceived stigma and consumer satisfaction. *Prev. Sci.* **7,** 167–177.
13. Nation, M., Crusto, C., Wandersman, A., Kumpfer, K.L., Seybolt, D., Morrissey-Kane, E., and Davino, K. (2003) What works in prevention: principles of effective prevention programs. *Am. Psychol.* **58,** 449–456.
14. Catalano, R.F., Berglund, M.L., Ryan, J.A.M., Lonczak, H.S., and Hawkins, J.D. (2002) Positive youth development in the United States: research findings on evaluations of positive youth development programs. *Prev. Treat.* **5**, Article 15.
15. Seligman, M.E.P. and Csikszentmihalyi, M. (2000) Positive psychology. *Am. Psychol.* ***55*(1)**, 5–14.
16. Amodeo, M. and Collins, M.E. (2007) Using a positive youth development approach in addressing problem-oriented youth behavior. *Fam. Soc*. **88(1)**, 75–85.
17. Shek, D.T.L. (2006) Construction of a positive youth development program in Hong Kong. *Int. J. Adolesc. Med. Health* **18(3),** 299–302.
18. Shek, D.T.L., Lee, T.Y., Sun, R.C.F., and Lung, D.W.M. (2008) Positive youth development programs targeting students with greater psychosocial needs: subjective outcome evaluation. *TheScientificWorldJOURNAL:* TSW Holistic Health & Medicine **8,** 73–82.
19. Shek, D.T.L. and Sun, R.C.F. (2008) Helping adolescents with greater psychosocial needs: evaluation of a positive youth development program. *TheScientificWorldJOURNAL:* TSW Holistic Health & Medicine **8,** 575–585.
20. Shek, D.T.L., Siu, A.M.H., Lui, J.H.Y., and Lung, D.W.M. (2006) *P.A.T.H.S. to Adulthood: A Jockey Club Youth Enhancement Scheme (Evaluation Manual).* Social Welfare Practice and Research Centre, The Chinese University of Hong Kong.
21. SPSS for Windows (2009) Statistical Package for the Social Sciences. SPSS Inc., Chicago.
22. Schon, D.A. (1991) *The Reflective Practitioner: How Professionals Think in Action.* Basic Books, New York.
23. Cheung C.K. (2006) Experiential learning strategies for promoting adolescents' voluntarism in Hong Kong. *Child Youth Care For*. **35(1),** 57–78.
24. Teresa, B.F. and Hinkle, J.S. (2002) Adventure based counseling: an innovation in counseling. *J. Couns. Dev*. **80,** 277–285.
25. Moote, G.T., Jr. and Wodarski, J.S. (1997) The acquisition of life skills through adventure-based activities and programs: a review of the literature. *Adolescence* **32(125),** 143–167.
26. Lee, T.Y., Shek, D.T.L., and Kwong, W.M. (2007) Chinese approaches to understanding and building resilience in at-risk populations. *Child. Adolesc. Psychiatr. Clin. N. Am*. **16,** 377–392.
27. Wong, K.Y. and Lee, T.Y. (2005) Professional discourse among social workers working with at-risk adolescents in Hong Kong: risk or resilience? In *Pathways to Resilience: a Handbook of Theory, Methods, and Intervention.* Ungar, M., Ed. Sage, Thousand Oaks, CA. pp. 313–327.
28. Leming, J.S. (2001) Integrating a structured ethical reflection curriculum into high school community service experiences: impact on students' sociomoral development. *Adolescence* **36,** 33–45.
29. Scales, P.C., Blyth, D.A., Berkas, T.H., and Kiesmeier, J.C. (2000) The effects of service learning on middle school students' social responsibility and academic success. *J. Early Adolesc.* **20,** 332–358.
30. Center for Substance Abuse Prevention (1996) A Review of Alternative Activities and Alternatives Programs in Youth-Oriented Prevention. Washington, D.C.
31. Hawkins, J.D. and Catalano, R.F. (1992) *Preparing for the Drug-Free Years*. Jossey-Bass, San Francisco.

32. Janz, N.K., Zimmerman, M.A., Wren, P.A., and Israel, B.A. (1996) Evaluation of 37 AIDS prevention projects: successful approaches and barriers to program effectiveness. *Health Educ. Q.* **23,** 80–97.
33. Park, N. and Peterson, C. (2003) Early intervention from the perspective of positive psychology. *Prev. Treat.* **6,** Article 35.
34. Nixon, R. (1997) Introduction. *Child Welfare* **76(5),** 571–575.
35. Erikson, E. (1968) *Identity, Youth and Crisis.* Norton, New York.
36. Law, B.M.F. and Shek, D.T.L. (2009) Beliefs about volunteerism, volunteering intention, volunteering behavior, and purpose in life among Chinese adolescents in Hong Kong. *TheScientificWorldJOURNAL:* TSW Child Health & Human Development **9,** 855–865.
37. Dodge, K.A., Dishion, T.J., and Lansford, J.E. (2006) Deviant peer influences in intervention and public policy for youth. *Soc. Policy Rep*. **20(1)**, 3–19.
38. Gingiss, P.M., Roberts-Gray, C., and Boerm, M. (2006) Bridge-it: a system for predicting implementation fidelity for school-based tobacco prevention programs. *Prev. Sci.* **7**, 197–207.
39. Pind, J., Gunnarsdóttir, E.K., and Jóhannesson, H.S. (2003) Raven's standard progressive matrices: new school age norms and a study of the test's validity. *Pers. Individ. Dif.* **34,** 375–386.
40. Shek, D.T.L. Subjective outcome and objective outcome evaluation findings: insights from a Chinese context. *Res. Soc. Work Pract.*, in press.
